# Prevalence and Clinical Risk Factors of Thyroid Cancer in Retrosternal Goiter: A Retrospective Comparative Study with Cervical Multinodular Goiter

**DOI:** 10.3390/jcm14020489

**Published:** 2025-01-14

**Authors:** Francesco Paolo Prete, Giuseppe Massimiliano De Luca, Lucia Ilaria Sgaramella, Alessandro Pasculli, Giovanna Di Meo, Carlotta Testini, Giuliana Rachele Puglisi, Matteo Rugge, Angela Gurrado, Mario Testini

**Affiliations:** 1 Academic General Surgery Unit “V. Bonomo”, Department of Precision and Regenerative Medicine and Jonian Area (DiMePRe-J), University of Bari “Aldo Moro” Medical School, 70124 Bari, Italyilaria.sgaram@gmail.com (L.I.S.); pascullialessandro@gmail.com (A.P.); mario.testini@uniba.it (M.T.); 2 Academic Imaging Diagnostic Unit, Interdisciplinary Department of Medicine, University of Bari “Aldo Moro” Medical School, 70124 Bari, Italy

**Keywords:** retrosternal goiter, mediastinal goiter, multinodular goiter, thyroid cancer, unexpected differentiated thyroid carcinoma, fine-needle aspiration

## Abstract

**Background:** Surgical intervention in asymptomatic retrosternal goiter (RSG) is debated in the absence of suspicious cytology, while performing fine-needle aspiration (FNA) is challenging in thyroids with mediastinal extension. The rate of unexpected thyroid cancers found at the time of thyroidectomy varies widely, while the notion of increased cancer incidence in RSG with respect to cervical goiters is still controversial. **Methods:** We retrospectively reviewed 411 patients with a preoperative diagnosis of multinodular goiter (MNG) (114 retrosternal, 297 cervical) who underwent thyroidectomy at an academic endocrine surgery referral center between January 2019 and October 2022. Rates of cancer detected on the final pathology examination, overall and not anticipated by preoperative workup, were compared between cervical MNG (cMNG) and RSG. **Results:** Patients with RSG were older (58.2% vs. 50.6%; *p* < 0.001) and more likely to be men (34.2% vs. 23%; *p* = 0.014). Overall, thyroid cancer was found in 49.5% of cMNG specimens and in 35.1% of RSGs (*p* = 0.02), and cancer > 1 cm was found in 37.4% of cMNG and 17.4% of RSG (*p* = 0.003). Prevalence of unexpected thyroid cancer was not significantly different between cMNGs (29.8%) and RSGs (28.8%). Unexpected carcinomas > 1 cm impacted 11% of all RSGs. **Conclusions:** In this study, the prevalence of unexpected thyroid cancer in RSG was similar to cMNG and significant from a clinical standpoint, with 1 in every 10 RSG diagnosed with differentiated cancer > 1 cm. Findings may be partially attributed to the difficulty in performing FNA in the mediastinum. Surgeons should counsel patients preoperatively regarding the risk of unexpected thyroid cancer to set appropriate expectations of outcome.

## 1. Introduction

Intrathoracic, cervicothoracic, subclavicular, mediastinal, substernal or retrosternal goiter (RSG) are all terms used to define an enlarged thyroid gland that grows inferiorly and passes through the thoracic inlet into the thoracic cavity, as opposed to cervical goiter which remains within the neck area [1,2,3,4]. The most frequent etiology of RSG is multinodular goiter (MNG), as RSG tends to develop functional autonomy and multinodularity in its natural evolution. RSG is not very well defined as an entity, as there is no unanimity concerning the amount of thyroid gland that should be located in the thorax, or at what level it is located; depending on the criteria used, there is an uneven incidence of RSG in different series, with a rate that ranges from 0.2% to 46% of the total number of goiters [1,2,3,4]. Thyroidectomy is the treatment of choice for RSG, and indications generally are the inefficacy of medical treatment, compression symptoms or airway obstruction and hyperthyroidism or malignancy, while the concern with thyroidectomy in RSGs is the potential for higher morbidity, including hypoparathyroidism, laryngeal nerve damage and even higher risk of mortality in the case of a thoracic approach [1,3,4]. Because of these risks, the role of surgical intervention is debated in asymptomatic RSGs, which are increasingly detected incidentally through imaging investigations [3,5]. Proponents of thyroidectomy argue that asymptomatic RSG is a risk for thyroid cancer [6,7]. Suspicion of thyroid cancer in RSG is currently based on clinical, imaging or fine-needle aspiration cytology (FNA) findings [1,2,8,9]. However, performing FNA in patients with thyroids extending to the mediastinum is challenging [9,10], so consideration of potential malignancy harbored by RSG may rely on existing knowledge of prevalence [11,12]. RSGs have been historically associated with a higher thyroid cancer incidence than cervical ones [13,14], though this notion is still controversial; thyroid cancer may be found in a widely varying proportion of resected MNG specimens, 3–35%, but few studies differentiate the prevalence of thyroid cancer between cervical MNG (cMNG) and RSG, and when they do so multiple definitions of RSG have to be considered [4,11,14,15]. This study investigates the comparative prevalence of thyroid cancer in post-thyroidectomy specimens of cervical MNG (cMNG) and RSG defined using Katlic’s definition [16], along with the impact on cancer prevalence of patient- and disease-related clinical factors.

## 2. Materials and Methods

A single-center, retrospective study was conducted at the Academic Unit of General Surgery “V. Bonomo” of Policlinico di Bari University Hospital, answering the following research question: in patients who underwent thyroidectomy for cMNG or RSG, what was the comparative frequency of thyroid carcinoma that was found at the pathology exam, overall and not anticipated by preoperative workup?

In the interval period from January 2019 to October 2022, out of 621 surgical procedures conducted on the thyroid gland, 411 thyroidectomies performed in adult patients with MNG, either cervical or retrosternal, were identified to be included in this study through a review of the institutional prospective endocrine surgery database. Exclusion criteria were lateral neck dissection, laryngectomy plus thyroidectomy, other cancers spreading into the thyroid, Graves’ disease with diffuse goiter, minimally invasive video-assisted thyroidectomy [17] and uninodular thyroid disease.

MNG was defined as an enlargement of the thyroid gland in the context of which at least two nodules were present in one thyroid lobe.

MNG with retrosternal extension, or RSG, was defined as more than 50% of the gland, or of any thyroid nodule, being located in the mediastinum with the patient’s neck extended when lying on the operating table [16,18].

According to this definition, 114 RSG and 297 cervical MNGs (cMNG) were identified, composing the two groups of this study.

### 2.1. Study Protocol

As per institutional protocol, thyroid function tests and cervical US were routinely performed prior to surgery. FNA on suspicious nodules was indicated and performed as defined in the revised 2016 ATA guidelines [19]. Preoperative cytology was reviewed by a board-certified pathologist. Computed tomography with multiplanar reformatting was used according to the surgeon’s judgement to evaluate the mediastinal portion of the gland [20].

### 2.2. Measures of Outcome

In patients undergoing thyroidectomy for retrosternal or cervical MNG, we aimed to do the following:

- Comparatively assess the prevalence of overall and unexpected thyroid cancer (primary);
- Investigate the relationship between clinico-pathological variables and the occurrence of thyroid malignancy in RSGs (secondary).

To proceed to comparative analysis for each of the two groups, RSG and cMNG, we assessed the following:

- The overall prevalence of thyroid cancer, defined as the number of cases of thyroid cancer found in the pathology exams over the total number of cases;
- The overall prevalence of unexpected thyroid cancer, defined as the number of cases of thyroid cancer that were diagnosed at the postoperative pathology exam stage and which were not anticipated by preoperative diagnostic workup, over the total number of cases;
- The rate or fraction the cases of thyroid cancer > 1 cm diameter diagnosed among thyroid cancers overall and among unexpected thyroid cancers, respectively;
- Univariable and multivariable analyses of factors associated with cancer frequency, conducted respectively for all MNG (cMNG and RSG) and for RSG only.

Thyroid cancer histological types and FNA cytology classes, according to The Italian Consensus for the Classification and Reporting of Thyroid Cytology (ICCRTC) [21], were collected. Microcarcinomas and cancers > 1 cm were considered for the final interpretation of the data.

To analyze the relationship between patient and disease-related characteristics and the finding of thyroid cancer at the post-thyroidectomy pathology stage, we collected the following variables: age (in years), gender (male or female) and weight of the thyroid specimen (in grams). We also measured the rate of occurrence (number of cases over total period, per group) of the following variables: hyperfunction (toxic multinodular goiter), previous history of radiation exposure, family history of thyroid cancer, compression symptoms (dyspnea/orthopnea, voice changes and dysphagia), tracheal deviation, reintervention and total thyroidectomy.

### 2.3. Statistical Analysis

The data are presented as number of cases (%) or mean (range), as appropriate. Univariable associations between dichotomous and categorical outcome variables were examined using the Chi-square test/Fischer’s exact test as appropriate. The ANOVA Kruskal–Wallis’ test was used to compare differences in continuous variables between groups. To investigate the comparative rate of unexpected thyroid cancer, or cancer that was not anticipated by preoperative investigations, analysis was run excluding all cases where ultrasound-FNA features were documented or raised suspicion for thyroid cancer. The occurrence of thyroid cancer (of any type or size) was assessed in both cervical and retrosternal multinodular goiters in relation to preoperatively assessed disease and patient related characteristics using multivariable logistic regression analysis. Statistical analysis was conducted using SPSS^®^ver.26.0.0 software (IBM, Armonk, New York, NY, USA), with significance set at 0.05.

### 2.4. Ethical Considerations

This study was conducted in accordance with the Declaration of Helsinki. Written informed consent for the use of clinico-pathological data related to hospital admission was obtained from all patients included in the institutional prospective endocrine surgery database. Encrypted, de-identified data were stored in the database, and it was not possible to trace any of the data to actual individuals. Only information required for coherent description of cases was extracted. Data in electronic format were accessible to authorized personnel only. Individual patient consent was obtained for the use of clinical images.

## 3. Results

### 3.1. Demographics

The demographic characteristics of participants in the two groups are shown in Table 1. Patients with RSG were more frequently men and older with respect to cMNG. Respiratory symptoms, along with evidence of tracheal deviation, were also more significantly present in those with RSG.

### 3.2. Overall Prevalence of Thyroid Cancer

Data on the overall prevalence of thyroid cancer in the pathology specimen are presented in Table 2. We found that thyroid cancer, both overall and >1 cm diameter, was significantly more frequent in the cMNG specimens than in RSG. In particular, microcarcinoma accounted for approximately one fourth and half of all thyroid cancers found in cMNGs and RSGs, respectively. Differentiated thyroid cancer (DTC) was the most frequent cancer in both groups, of which papillary was the most frequently represented histotype.

### 3.3. Preoperative Suspicion of Thyroid Malignancy and Prevalence of Unexpected Thyroid Cancer

Table 3 shows the number of cases in which thyroid cancer was anticipated preoperatively for each of the study groups.

There were significant differences in patients’ access to FNA in the two groups, with cMNG being more frequently investigated with the aid of cytology. As a result of the preoperative workup, 1 in 3 of the cMNGs and 1 in 10 of the RSGs had preoperative information of a malignancy.

After excluding cases where preoperative workup was suggestive of thyroid cancer, analysis of prevalence showed no significant differences in the rate of unexpected thyroid cancer between RSG and cMNG (Table 4).

### 3.4. Univariable and Multivariable Analysis of Factors Associated with Cancer Frequency for MNGs in Any Location

Univariable analysis of patient- and disease-related clinical characteristics and findings of thyroid cancer in specimens across all 411 MNG included in this study are shown in Table 5. Younger age, compression symptoms (dyspnoea, dysphagia and voice changes), tracheal deviation and retrosternal extension were less frequently associated with the finding of malignancy in specimens with respect to benign nodules.

Upon multivariable analysis (Table 6), tracheal deviation was the only independent patient-related factor for decreased odds of finding thyroid cancer in the MNG (cervical and retrosternal) specimens.

### 3.5. Univariable and Multivariable Analysis of Factors Associated to Cancer Frequency for RSG Only

Univariable analysis found that a family history of thyroid cancer and male gender were significantly associated with thyroid cancer in RSG. Multivariable analysis (Table 7) showed that a family history of thyroid cancer and male gender were independent factors for thyroid cancer in RSG specimens, increasing the odds of finding cancer by 13.4 and 2.6 times, respectively.

## 4. Discussion

In this study, we investigated the frequency of thyroid cancer in the pathology specimens of patients with cervical and retrosternal MNG. Thyroid cancer has been found at a wide interval of 3 to 35% of surgically resected MNG specimens, and historically RSGs have been associated with higher thyroid cancer incidence than MNGs [4,9,14]. However, prevalence studies of thyroid cancer that differentiate cervical MNG and RSG are few [15].

We found that the overall cancer frequency in RSG, 35.1%, along with that of cancer > 1 cm diameters, 17.5%, were significantly lower than in cervical MNG. Previous evidence has shown that incidence of cancer in RSG is not higher than cMNG; a study by Sahbaz et al., which included in its analysis all cases of RSG irrespective of their preoperative FNA outcome, found that the frequency of DTC discovered at the pathology stage did not present significant differences between cMNG and RSG [22]. The results of a systematic review by White et al. also showed that incidence of DTC in RSG was not higher than in cervical goiters, and that the malignancy rate in RSG ranged between 3.7 and 22.6%. The majority of the studies included in this review did not specify tumor size, leaving uncertainty over the relative contribution of microcarcinomas to these figures [12]. In our study, cases of microcarcinoma were one of the main contributors to cancer frequency in RSG, as also previously found by Rios et al. [1].

Multiple factors are likely motivating variability in the incidence of cancer on RSG, including differing patterns of patient referral among centers, how RSG was defined in the first instance and the diagnostic capabilities and accuracy provided by FNA.

Different definitions result in wide oscillations in the frequency of RSG treated by thyroidectomy, which is between 0.2% and 46%, depending on the amount of thyroid gland extending into the thorax, or at what level the lower border of the thyroid is located [1,2,3,4,5,6,16,18]. This study uses the commonly adopted Katlic’s definition, which is of practical importance as it is a useful predictor of sternotomy for the excision of RSG [4,16].

**Figure 1:**
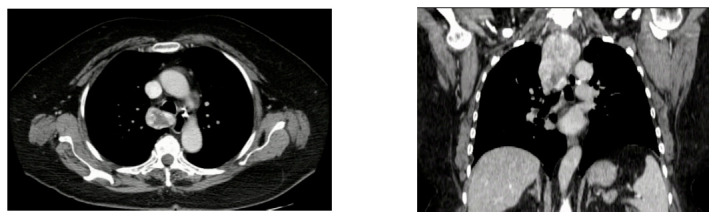
Axial and coronal CT scan of a patient with an 8 cm intrathoracic goiter not amenable to US-guided FNA.

Preoperative FNA was performed significantly more frequently in cMNG than in RSGs in patients from this study, as was also the case in a study by Campbell et al. [15]. Nodules located in the retrosternal components of goiters are not easily imaged by US due to the artefact generated by bony structures and so may remain unrecognized [23]. Intrathoracic nodules are often inaccessible to needle biopsy (Figure 1), but even when a target mediastinal thyroid lesion can be detected via US, FNA is usually discouraged due to the limited visibility and the possibility of causing damage to vital structures located in the mediastinum [24,25,26].

This makes the exclusion of malignancy particularly troublesome in RSG. Moreover, nodules < 1 cm are generally not biopsied, and even when US-guided FNA is performed in radiographically accessible suspicious lesions, it is possible that malignancy exists in alternative positions [11]. Computed tomography (CT) and/or magnetic resonance imaging (MRI), which provide a clear definition of the multidimensional size and morphology of the RSG, and may suggest infiltration into surrounding structures, are essential in surgical decision-making [14,27]. In specialized endocrine surgery units, intraoperative ultrasound may provide data on lymph node status, partially offsetting diagnostic limitations [28].

As in previous studies by Testini et al. and Campbell et al. [14,15], to comparatively assess the prevalence of unexpected thyroid cancer in RSG and cMNG, we performed analysis by excluding patients in whom preoperative FNA was suspicious for, or diagnostic of, malignancy. We found that the overall prevalence of unexpected thyroid cancer, around 29%, did not show significant differences between the RSG and cMNG groups. In a large multicenter study by Testini et al., DTC was unexpected in 23% of nearly 1000 cases of RSG, a prevalence not too distant from that of the present series, though in Testini et al. the prevalence was significantly higher than in cervical MNG, possibly a combined effect of referral patterns and varying definitions of RSG [14]. In Campbell et al., the 13.7% prevalence of unexpected DTC > 1 cm found during histological examination was also similar to the 11.5% observed in this study [15].

Previous studies found an association between compressive symptoms and the presence of cancer in MNG, both cervical and retrosternal [9]. Tracheal deviation, a radiological indicator of compression, was inversely related to the overall presence of thyroid cancer in this study. However, indicators of compression, such as symptoms of neck fullness, dysphagia or dyspnoea, do not necessarily predict thyroid cancer; both benign or malignant goiters may cause gradual, progressive symptoms as they grow larger.

We also found that, in RSG patients, a family history of thyroid cancer and male gender were independent factors for cancer in the thyroidectomy specimens. Male gender has been independently associated with malignancy in MNG in previous studies [9,29,30]; patients with a family history of thyroid cancer have also been found significantly more likely to have cancer diagnosed via the MNG specimen [23]. However, most cancers encountered in this study did not have obvious predisposing features, and lack of risk factors alone cannot ensure the benign nature of the goiter.

Limitations of this study include its retrospective design and the uniqueness of the patient subset, as cancer rates depend on the patients selected to undergo surgical resection; a fraction of patients in this study, around 15% in both groups, did not undergo total thyroidectomy, so in these cases the remaining thyroid tissue could not be assessed, limiting the contribution of these cases to unexpected thyroid cancer frequencies. In this study, the location of a cancerous nodule within the thyroid gland could not be consistently mapped onto the retrosternal portion of the gland, as the pathologist did not always know where the retrosternal portion began. The findings of thyroid cancer prevalence may also reflect different approaches to the pathological processing of surgical specimens. The location (cervical or mediastinal) of suspect neoplastic nodules within an RSG likely dictated whether FNA could be performed, as mediastinal nodules were not sampled. Finally, microcarcinomas might not be all clinically insignificant (e.g., > 55 yrs, the presence of positive nodes and tumor biology).

The strength of this study is the inclusion of a large series of participants, where prevalence of thyroid cancer has been examined as closely as possible to true clinical scenarios, in both cervical and retrosternal MNG, presenting also the impact of FNA on prevalence. To our knowledge, this is the first study that investigates the prevalence of thyroid cancer in RSG both before and after FNA is considered, so the results may be applicable to discussion of surgery with both patients with RSG who had or did not have FNA of their nodules. Multivariable analysis was also run to control for confounding factors. Moreover, the results obtained appear well comparable to previously published series [27].

**Figure 2:**
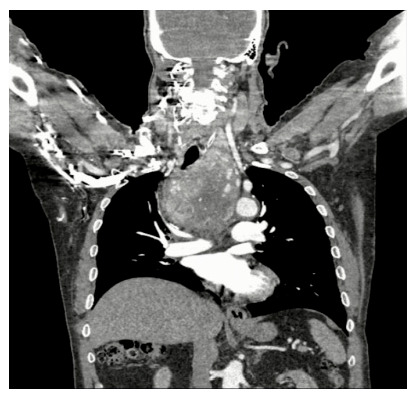
Contrast-enhanced CT scan of a patient with large 12 cm-intrathoracic goiter with calcifications. Postoperative pathology revealed poorly differentiated papillary thyroid carcinoma.

As per the data provided from this study, there is no convincing evidence that the incidence of malignancy in RSG is higher compared to cMNGs as previously represented [14,15]. Despite this, the overall prevalence of thyroid cancer in patients with RSG is clinically significant, as more than 1 in 4 patients with multiple nodules had unexpected thyroid cancer and, for cancer > 1 cm diameter, this was more than 1 in 10 patients, a figure that is in accordance with the published literature [15,29]. As documented in this study, most of these cancers represent differentiated thyroid carcinomas, but the possibility of aggressive subtypes should be also considered (e.g., poorly differentiated DTC—Figure 2) [31].

Recent studies show that perioperative complications in dedicated units present the same frequency in symptomatic and asymptomatic RSG [27,32,33,34]. An assessment of post-thyroidectomy complications was beyond the scope of this study, though in recently published evidence from this institution, RSG was not found to be an independent risk factor for postoperative complications or for increased complexity of thyroidectomy [34]. However, the extent and type of retrosternal extension and availability of expertise, as well as dedicated facilities in specialized units, may impact postoperative complications [35].

On the basis of the results from this study and the available evidence from the literature, we conclude that the risk of unexpected cancer in RSG should be enough to recommend thyroidectomy in all asymptomatic goiters.

Not all patients may be suitable candidates for thyroidectomy: in asymptomatic older adults or frail patients, life expectancy, the capacity to withstand potential complications from thyroidectomy for RSG and the availability of a specialized unit should be considered prior to surgery [34,35,36,37,38,39,40]. In this respect, evidence from this study can be used to balance the benefits and risks of thyroidectomy in subjects at significant risk for postoperative complications, and to help counsel patients prior to surgery on the risk of unexpected thyroid cancer to set appropriate expectations of outcome.

## 5. Conclusions

In this study, the overall rate of postoperatively discovered thyroid cancer in RSGs was lower than that in cervical MNGs. This may be attributed to the lower rate of FNAs performed in RSGs compared to cervical MNGs. When FNA was not factored in, the rate of unexpected thyroid cancer appeared similar between cMNGs and RSGs. Cancer prevalence in RSGs was significant from a clinical standpoint, with over 1 in 10 patients having had unexpected DTC > 1 cm found at pathology exam stage, enough to indicate thyroidectomy in asymptomatic retrosternal goiters. Surgeons should counsel patients prior to surgery regarding the risk of unexpected thyroid cancer to set appropriate expectations of outcome.

## Figures and Tables

**Table 1:** Demographics.

Demographics	Substernal Goiter (*n* = 114)	Cervical Goiter (*n* = 297)	*p* Value
Gender M—*n* (%)	39 (34.2)	68 (22.9)	0.014
Mean age—years (range)	58.2 (22–90)	50.6 (18–81)	<0.001
Toxic multinodular goiter	13 (11.4)	38 (13.2)	0.376
Radiation exposure	0 (0)	2 (0.7)	0.522
Family history of thyroid ca	6 (5.3)	32 (10.8)	0.084
Dysphagia	34 (29.8)	82 (27.6)	0.370
Dyspnea/orthopnea	28 (24.6)	31 (18.4)	<0.001
Voice changes	19 (16.7)	40 (13.5)	0.248
Any symptoms	35 (30.7)	88 (29.7)	0.468
Tracheal deviation	50 (43.9)	49 (16.6)	<0.001
Reintervention	4 (1.3)	2 (1.8)	0.531
Total thyroidectomy	98 (86)	254 (85.5)	0.524

**Table 2:** Prevalence of thyroid cancer. RSG: retrosternal goiter; cMNG: cervical multinodular goiter.

Final Pathology, All Cases	RSG (*n* = 114)	cMNG (*n* = 297)	*p* Value
Total mass (weight, grams)	114.45 (22–300)	37.7 (10–140)	<0.001
Thyroid cancer (All)	40 (35.1)	147 (49.5)	0.006
Thyroid cancer > 1 cm	20 (17.5)	111 (37.4)	<0.001
Papillary	12 (60)	98 (88.3)	
Follicular	4 (20)	9 (8.1)	
Medullary	1 (5)	2 (1.8)	
Poorly diff/Anaplastic	2 (10)	2 (1.8)	
Papillary microcarcinoma	20 (17.5)	36 (12.1)	0.103

**Table 3:** FNA cytology performance and preoperative suspicion of malignancy. Cytology classification: The Italian Consensus for the Classification and Reporting of Thyroid Cytology—ICCRTC; RSG: retrosternal goiter; cMNG: cervical multinodular goiter.

Fine Needle Aspiration Frequency and Outcome	RSG (*n* = 114)	cMNG (*n* = 297)	*p* Value
FNA n. (%)	36 (31.6)	140 (47.1)	0.003
Inadequate	2 (1.8)	2 (0.7)	
Benign	18 (15.8)	25 (8.4)	
Indeterminate	6 (5.3)	15 (5.1)	
Suspicious/malignant (TIR 3B-4-5)	10 (8.8)	99 (33.3)	
Preoperative suspicion of malignancy (Doppler US, FNA, Pt history)	10 (8.8)	106 (35.7)	<0.001

**Table 4:** Prevalence of unexpected thyroid cancer. RSG: retrosternal goiter; cMNG: cervical multinodular goiter.

Final Pathology, Cases with No Preoperative Suspicion of Cancer	RSG (*n* = 104)	cMNG (*n* = 191)	*p* Value
Total mass (weight, grams)			
Thyroid cancer (All)	30 (28.8)	57 (29.8)	0.484
Thyroid cancer > 1 cm	12 (11.5)	34 (17.8)	0.104
Papillary	7 (58.3)	28 (82.4)	
Follicular	3 (25)	5 (14.7)	
Medullary	0	0	
Poorly diff/Anaplastic	2 (16.7)	1 (2.9)	
Papillary microcarcinoma < 1 cm	18 (17.3)	23 (12)	0.142

**Table 5:** Univariable analysis of clinico-pathological variables and cancer occurrence among all MNG (cervical and retrosternal) in this study.

Univariable Analysis	Benign (*n* = 224)	Malignant (*n* = 187)	*p* Value
Age (years, +/−SD)	53.8 +/−14.6	50.7 +/−14.4	0.039
Total mass (weight, grams)	65 +/−52.5	49.5 +/−57	0.207
Male gender	54 (24.1)	53 (28.3)	0.330
Compression symptoms (any)	80 (35.7)	43 (23)	0.005
Tracheal deviation	69 (30.8)	30 (16.1)	0.001
Retrosternal extension	74 (33)	40 (21.4)	0.009
Radiation exposure	1 (0.4)	2 (1.1)	0.460
Toxic goiter	33 (15.4)	18 (9.6)	0.082

**Table 6:** Multivariable analysis of factors for thyroid cancer finding at pathology exam, all operated MNG (cervical and retrosternal).

Thyroid Cancer on Specimen, All Cases (cMNG and RSG)	Exp (B)	95%CI for Exp (B)	*p* Value
Tracheal deviation	0.477	0.237–0.958	0.038

**Table 7:** Multivariable analysis of factors for thyroid cancer finding at pathology in RSG.

Thyroid Cancer on RSG Pathology	Exp (B)	95%CI for Exp (B)	*p* Value
Family history of thyroid cancer	13.424	1.409–127.933	0.024
Male gender	2.575	1.098–6.036	0.030

## Data Availability

The data are available on request from the corresponding author.
